# Role of MRI in Breast Cancer Staging: A Case-Based Review

**DOI:** 10.7759/cureus.20752

**Published:** 2021-12-27

**Authors:** Swati Sharma, Fiorella G Vicenty-Latorre, Sherif Elsherif, Smita Sharma

**Affiliations:** 1 Radiology, University of Florida College of Medicine, Jacksonville, USA

**Keywords:** review, role, mri, staging, breast cancer

## Abstract

Breast cancer is the most commonly diagnosed cancer in women in the United States and is also the second leading cause of cancer deaths in women. The five-year survival rate and overall prognosis are largely dependent on the stage at diagnosis. Our article highlights the role of magnetic resonance imaging (MRI) in breast cancer staging and updates to the American Joint Committee on Cancer (AJCC) 8th edition guidelines regarding breast cancer staging that are relevant to radiologists. It provides a case-based approach to emphasize the key findings that the radiologist should report on a breast MRI to aid the clinicians in staging and management.

## Introduction

Breast cancer is the second most commonly diagnosed cancer worldwide, and the incidence is higher in more industrialized countries. It is the most diagnosed cancer in women in the United States and is the second leading cause of cancer deaths in women. Approximately 250,000 new breast cancer cases were reported in 2016. Both the five-year survival rate and overall prognosis are dependent on staging.

The clinician determines the pre-operative stage of breast cancer by combining the physical examination and radiographic findings. Radiologists should be familiar with the tumor, node, and metastasis (TNM) staging system so that they can convey the most pertinent findings to the clinician. This article discusses the TNM staging system and reviews the role of MRI in accurate breast cancer staging. Although contrast-enhanced breast MRI is not a replacement for mammography and ultrasound, it is an important modality in staging breast cancer [1]. MRI better defines the tumor extent, local spread to the skin and nipple, and nodal involvement. It can identify additional tumor sites in 15-27% of patients in the same breast and 3-10% of patients in the contralateral breast [2,3]. This article aims to highlight the usefulness of MRI in breast cancer staging as well as to briefly discuss its limitations.

According to the American College of Radiology (ACR) practice parameters, the current indications for MRI in breast cancer are as follows [4]:

Screening - (1) for screening and surveillance of high-risk patients (women with greater than or equal to 20% lifetime risk of breast cancer, for example, individuals with genetic predisposition to breast cancer as determined by either gene testing or family pedigree, including breast cancer susceptibility genes 1 and 2 mutations), (2) may be considered as a screening supplement to mammogram for intermediate-risk patients, (3) may be considered for patients with newly diagnosed breast malignancy to detect occult malignancy in the contralateral breast, and (4) may be considered for patients with breast augmentation with silicone or saline implants and/or silicone injections in whom mammogram is difficult.

The extent of disease - (1) for assessment of disease extent, multifocality, multicentricity in invasive ductal carcinoma and ductal carcinoma in situ, for preoperative assessment of biopsy-proven breast cancer, (2) may be useful to evaluate invasion deep to fascia, (3) may be useful for subsequent surgical planning for post-lumpectomy patients with positive margins, and (4) for assessment of treatment response before, during, and/or after chemotherapy.

Additional evaluation - (1) for assessment of suspected recurrence of cancer with an inconclusive initial evaluation, (2) for metastatic cancer when the primary cancer is unknown and suspected to be of breast origin, (3) for lesion characterization of indeterminate lesions on initial imaging, (4) for bloody nipple discharge with normal mammogram and ultrasound, (5) for postoperative tissue reconstruction if recurrence of cancer is suspected, and (6) for MRI-guided biopsy.

The American Society of Breast Surgeons does not recommend the use of routine diagnostic MRI in all patients with newly diagnosed breast cancer. However, it supports the use of MRI in select situations: (1) to look for occult breast cancer in patients with Paget’s disease of the nipple or axillary node metastasis, when the initial workup has not identified a primary breast cancer; (2) to assess the extent of cancer in patients at high risk for additional disease, including those with invasive lobular carcinoma, extremely dense breasts, or discrepancies between physical exam and imaging findings; and (3) to evaluate the patient’s eligibility and response to neoadjuvant endocrine therapy or chemotherapy and aid in surgical decision-making.

## Materials and methods

We conducted a retrospective chart review of up to 15 charts of female patients within our hospital system who had known biopsy-proven breast cancer and had undergone breast MRI. The charts were identified within the software database for radiology reports using the search terms “breast cancer” and “breast MRI.” We selected the most representative cases for every TNM stage based on the imaging features. In this article, the key images of these selected cases, including multiple data points from MRI, relevant clinical history, and biopsy reports, are used to highlight the role of MRI in breast cancer staging.

At our institute, breast MRI is performed with either a 1.5-Tesla or a 3-Tesla magnet using breast coils. The protocol includes a non-fat-saturated T1-weighted sequence, a short tau inversion recovery sequence, a fat-saturated T1-weighted sequence, dynamic contrast-enhanced sequences approximately 60 seconds to six minutes after contrast administration, and maximum intensity projection image.

Breast cancer staging

The American Joint Committee on Cancer (AJCC) is one of the major bodies governing the guidelines for cancer staging. For this study, we used the 8th and latest edition for breast cancer staging that became effective in the United States as of January 1, 2018 (Tables 1-5). This version incorporates the use of biomarkers in countries where they are widely available to designate prognostic staging. Radiologists should be aware that this will not affect anatomic TNM staging [5-7].

**Table 1:** Summary of changes in the AJCC 8th edition AJCC = American Joint Committee on Cancer; M = metastasis; N = nodes; pN = pathologic N; T = tumor; TNM = tumor, node, and metastasis; HER2 = human epidermal growth factor receptor-2.

Changes in tumor classification (T)	Lobular carcinoma in situ is classified as a benign entity and is removed from TNM staging. Multiple cancers are documented using (m) modifier. Satellite tumor nodules in the skin must be separate from the main mass to qualify as T4b. Tumors measuring >1 mm but <2 mm should be rounded to 2 mm.
Measurement of the metastatic lymph node on pathology (N)	Pathologic staging of lymph nodes uses only the largest contiguous tumor deposit for pN
Definition of distant metastasis (M)	pM0 is not a valid category, cM0, cM1, and pM1 are now used
Clarification of post neoadjuvant therapy staging	ycTNM and ypTNM will be used after therapy. Response to therapy is evaluated as complete response, partial response, or no response
Adoption of prognostic staging (clinical and pathologic prognostic stages)	Inclusion of biomarkers: tumor grade, hormone receptors, and HER2 (in countries where these are widely available). Inclusion of multi-gene panels. The clinical prognostic stage is assigned regardless of the type of therapy received. Pathologic prognostic stage will be assigned to patients who underwent surgery as initial treatment (excludes patients who received neoadjuvant therapy)

**Table 2:** T descriptors for TNM staging of breast cancer TNM = tumor, node, and metastasis.

Descriptor	Definition
TX	Primary tumor cannot be assessed
T0	No evidence of primary tumor
Tis (DCIS)	Ductal carcinoma in situ
Tis (Paget’s)	Paget’s disease of nipple unrelated to invasive carcinoma or DCIS in underlying breast parenchyma
T1	Tumor ≤20 mm
- T1mi	Tumor ≤1 mm in greatest dimension
- T1a	Tumor >1 mm but ≤5 mm
- T1b	Tumor >5 mm but ≤10 mm
- T1c	Tumor >10 mm but ≤20 mm
T2	>20 mm to ≤50 mm
T3	>50 mm
T4	Tumor of any size with direct extension to the chest wall and/or skin - not dermis only
- T4a	Chest wall invasion
- T4b	Ulceration or edema (including peau d’orange) of less than one-third of the breast, macroscopic satellite tumor nodules on the skin
- T4c	Presence of both T4a and T4b
- T4d	Inflammatory carcinoma

**Table 3:** N descriptors for TNM staging of breast cancer TNM = tumor, node, and metastasis.

Descriptor	Definition
NX	Regional lymph nodes cannot be assessed
N0	No regional lymph node metastasis
N1	Metastasis to movable ipsilateral level I-II axillary lymph nodes
N2	Metastasis to ipsilateral level I-II axillary lymph nodes that are matted, or in clinically detected ipsilateral internal mammary nodes in the absence of clinically evident axillary lymph nodes metastasis
- N2a	Metastasis to ipsilateral level I-II axillary lymph nodes matted to one another or other structures
- N2b	Metastasis only in clinically detected ipsilateral internal mammary nodes in the absence of clinically evident level I-II axillary lymph node metastasis
N3	Metastasis in ipsilateral infraclavicular (level III) lymph nodes with or without level I-II axillary lymph node involvement or ipsilateral internal mammary with ipsilateral level I-II axillary lymph nodes or ipsilateral supraclavicular lymph nodes with or without axillary or internal mammary lymph node involvement
- N3a	Metastasis in ipsilateral infraclavicular lymph nodes
- N3b	Metastasis in ipsilateral axillary and internal mammary lymph nodes
- N3c	Metastasis in ipsilateral supraclavicular lymph nodes

**Table 4:** M descriptors for TNM staging of breast cancer TNM = tumor, node, and metastasis.

Definition	Descriptor
M0	No clinical or radiographic evidence of distant metastasis
- cM0( i+)	No clinical or radiographic evidence of distant metastasis, but deposits of molecularly or microscopically detected tumor cells in circulating blood, bone marrow, or other non-regional nodal tissue that are no larger than 0.2 mm in a patient without symptoms or signs of metastasis
cM1	Distant detectable metastasis as determined by classic clinical and radiographic means or histologically proven to be larger than 0.2 mm

**Table 5:** AJCC summary of anatomic stage groups for breast cancer AJCC = American Joint Committee on Cancer.

Stage	Tumor	Node	Metastasis
0	Tis	N0	M0
IA	T1	N0	M0
IB	T0	N1mi	M0
	T1	N1mi	M0
IIA	T0	N1	M0
	T1	N1	M0
	T2	N0	M0
IIB	T2	N1	M0
	T3	N0	M0
IIIA	T0	N2	M0
	T1	N2	M0
	T2	N2	M0
	T3	N1	M0
	T3	N2	M0
IIIB	T4	N0	M0
	T4	N1	M0
	T4	N2	M0
IIIC	Any T	N3	M0
IV	Any T	Any N	M1

Key parameters of MRI used in breast cancer staging

The key parameters of MRI used in breast cancer staging and highlighted in our article are described below.

Tumor Size

The primary tumor is usually mea­sured in three orthogonal dimensions, with the largest dimension used for staging purposes. If there is more than one malignant mass in the same breast, the size of the largest tumor should be used for staging purposes [1].

Multifocal and Multicentric Disease

Breast cancers are defined as multifocal when there is more than one distinct tumor within the same quadrant of the breast and multicentric when multiple cancers develop in different quadrants of the breast [8].

Pectoral Muscle Involvement

Pectoralis muscle involvement may be seen as muscle enhancement with obliteration of the fat plane between the tumor and muscle on MRI. However, obliteration of the fat plane as an isolated finding without associated muscle enhancement on MRI does not necessarily indicate muscle involvement [1].

Chest Wall Involvement

Contrast-enhanced breast MRI is the best imaging modality for determining the involvement of the chest wall. Direct invasion and involvement of the ribs, serratus ante­rior muscle, or intercostal muscles are considered chest wall involvement. According to the TNM staging system of the AJCC, involve­ment of the pectoralis major or minor muscle alone is not considered chest wall involvement [1].

Nodal Stage

Axillary lymph nodes are divided into levels I, II, and III. The level is based on the relationship between the lymph node and the pectoralis minor mus­cle. Level I lymph nodes are inferior to the inferolateral border of the pectoralis minor muscle, level II lymph nodes are posterior to and between the lateral and medial borders of the pectoralis minor muscle, and level III lymph nodes are medial to the superior border of the pectoralis minor muscle (including infraclavicular nodes) [1].

## Results

The representative cases compiled from our chart review are used to present a pictorial summary emphasizing the role of MRI in breast cancer staging. We have used the recognized descriptors from the American College of Radiology (ACR) standardized Breast Imaging Reporting and Data System (BI-RADS) MRI lexicon, as its consistent use allows for a more uniform descriptive terminology and can be used to dictate subsequent management recommendations [9].

TNM stage: 0 (TisN0M0)

**Figure 1:**
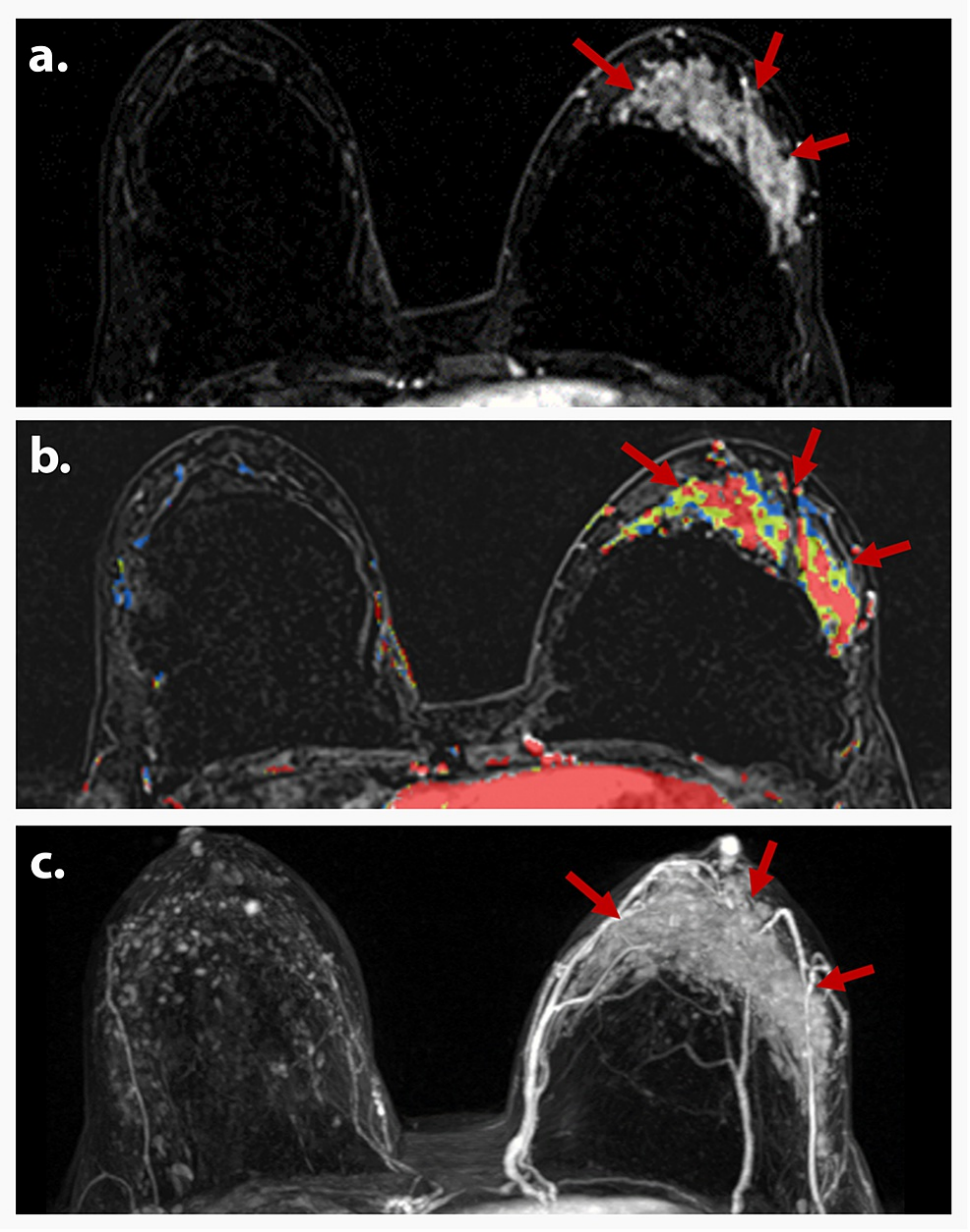
AJCC TNM stage 0 (TisN0M0) A 38-year-old premenopausal woman presented with a palpable left breast mass. The diagnostic mammogram was normal. Axial T1-weighted DCE (a), corresponding color-coded map (b), and MIP (c) magnetic resonance images show diffuse non-mass enhancement (red arrows) in the left breast with mixed kinetics. Biopsy depicted DCIS. Findings are consistent with AJCC TNM stage 0 (TisN0M0). AJCC: American Joint Committee on Cancer; TNM: tumor, node, and metastasis; DCE: dynamic contrast-enhanced; MIP: maximum intensity projection; DCIS: ductal carcinoma in situ.

In ductal carcinoma in situ (DCIS), breast MRI can show non-mass enhancement (Tis). The most commonly reported MRI manifestation of DCIS is clumped non-mass enhancement in a ductal, linear, segmental, or regional distribution. Ipsilateral axillary or any other lymphadenopathy (N0) is absent [10]. There will also be an absence of distant metastasis in the MRI field of view (M0). The extent of DCIS of the breast can assist in key surgical planning decisions between lumpectomy and mastectomy (Figure 1).

TNM stage: IA (T1N0M0)

**Figure 2:**
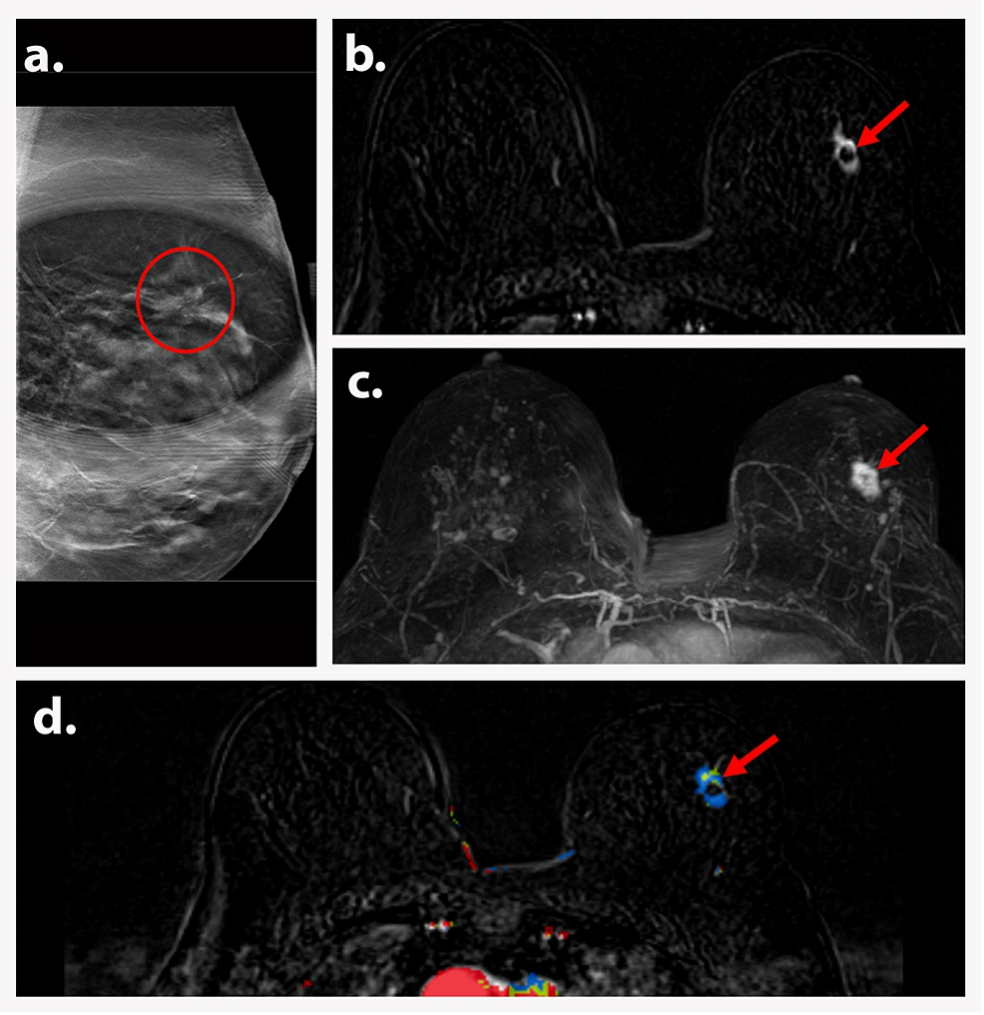
AJCC TNM stage IA (T1cN0M0) A 56-year-old postmenopausal woman presented with suspicious left breast mass on a screening mammogram. Left MLO spot compression view on a diagnostic mammogram (a) shows an irregular mass with spiculated margins (red circle) in the superior left breast. Axial T1-weighted DCE subtraction image (b) shows a 2 cm irregular enhancing mass (red arrow) with susceptibility artifact from biopsy clip without ipsilateral axillary lymphadenopathy. Axial MIP (c) and axial T1-weighted DCE with color (d) images demonstrate the irregular enhancing mass with persistent kinetics (red arrows). Biopsy depicted invasive carcinoma. Findings are consistent with AJCC TNM stage IA (T1cN0M0). AJCC: American Joint Committee on Cancer; TNM: tumor, node, and metastasis; MLO: mediolateral oblique; DCE: dynamic contrast-enhanced; MIP: maximum intensity projection.

In TNM stage IA, in addition to showing a tumor ≤20 mm (T1), MRI will show an absence of axillary lymphadenopathy (N0) and distant metastasis in the field of view (M0). Breast MRI assists with the accurate measurement of tumor size and therefore helps obtain an accurate assessment of T substaging (T1a-c) (Figure 2).

TNM stage: IB (T0-1N1miM0)

In TNM stage IB, MRI will demonstrate either no tumor or a tumor ≤20 mm (T0-1), without imaging-identifiable lymphadenopathy. However, in stage IB, microscopic metastasis in the ipsilateral axillary lymph nodes (N1mi) is present, which may be discovered during surgery and sentinel lymph node excision. There will also be an absence of distant metastasis in the MRI field of view (M0).

TNM stage: IIA (T0-1N1M0/T2N0M0)

**Figure 3:**
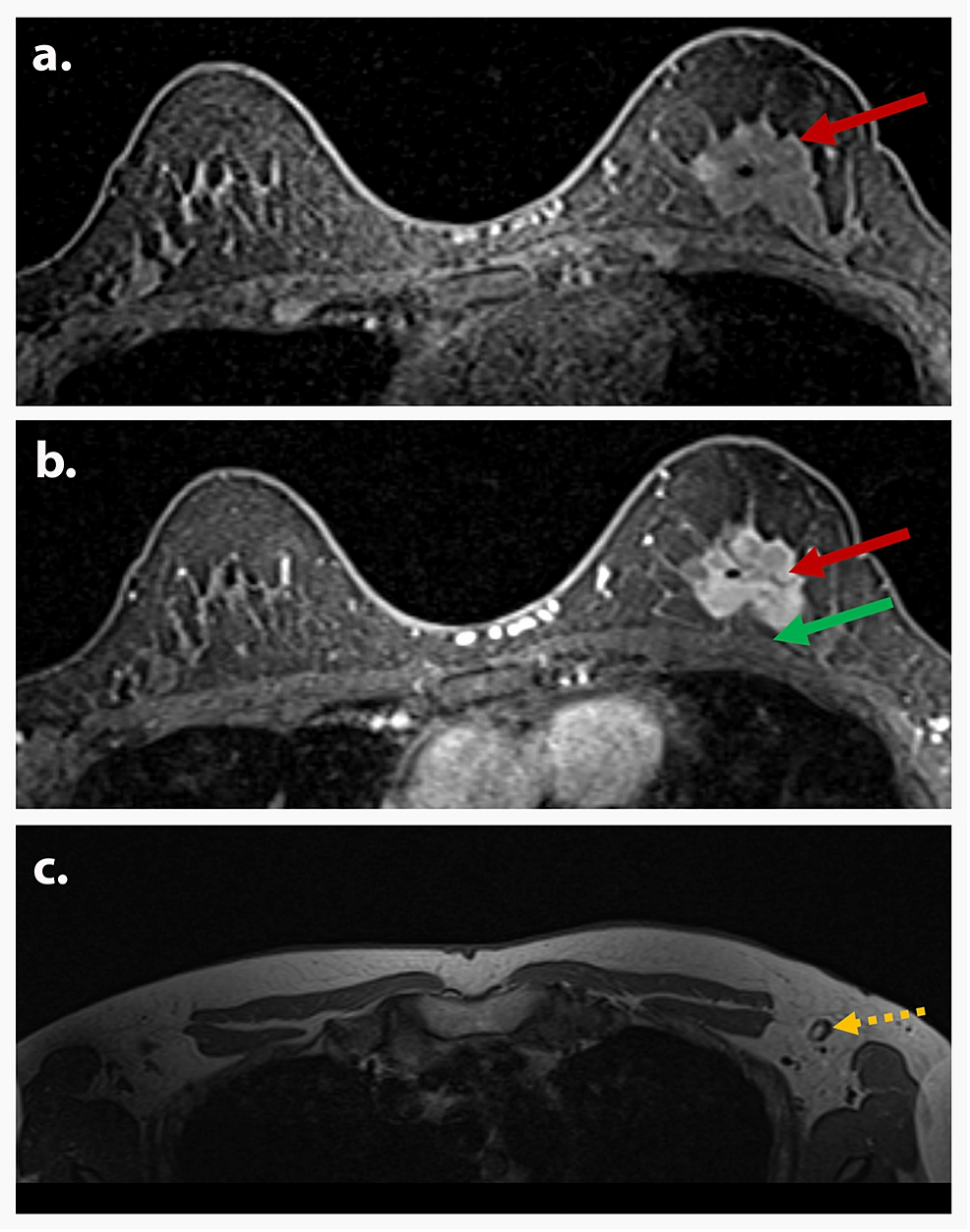
AJCC TNM stage IIA (T2N0M0) A 54-year-old presented with a left breast mass, which on evaluation with a diagnostic mammogram, ultrasound, and subsequent biopsy was proven to be invasive ductal carcinoma. Axial postcontrast T1-weighted subtraction MR image (a) shows an irregular heterogeneously enhancing posterior mass (red arrow) measuring 3.3 x 2.6 x 3.3 cm with spiculated margins in the upper central left breast, 12:00 position 3.9 cm from the nipple, with biopsy clip. Axial postcontrast T1-weighted MR image (b) demonstrates the irregular heterogeneously enhancing mass (red arrow) again with spiculated margins in the upper central left breast, with biopsy clip and without evidence of pectoral muscle infiltration (green arrow). Axial pre-contrast T1-weighted MR image (c) shows normal/physiologic non-enlarged axillary nodes (yellow arrow). Findings are consistent with AJCC TNM stage IIA (T2N0M0). AJCC: American Joint Committee on Cancer; TNM: tumor, node, and metastasis; MR: magnetic resonance.

In TNM stage IIA, MRI will demonstrate either no tumor or a tumor ≤20 mm (T0-1), with movable ipsilateral level 1/2 axillary lymphadenopathy (N1), or a tumor >20 mm but ≤50 mm (T2) without ipsilateral axillary lymphadenopathy (N0). There will be an absence of distant metastasis in the MRI field of view (M0) (Figure 3).

TNM stage: IIB (T2N1M0/T3N0M0)

**Figure 4:**
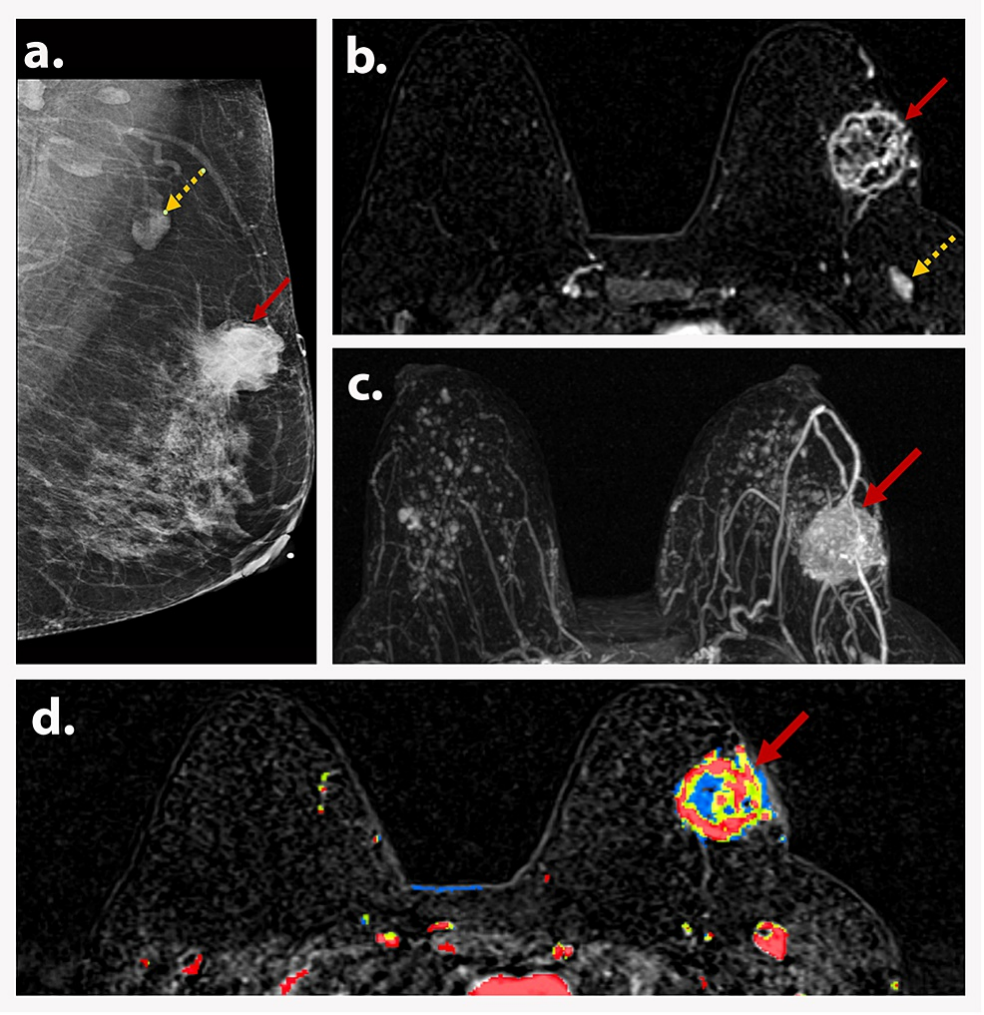
AJCC TNM stage IIB (T2N1M0) A 55-year-old postmenopausal woman presented with a palpable left breast mass. MLO view of the left breast (a) shows a 4 cm oval mass (red arrow) with spiculated and indistinct margins and suspicious axillary lymphadenopathy (dotted yellow arrow). Axial T1-weighted DCE subtraction image (b) shows a 3.9 cm irregular, spiculated, heterogeneously enhancing mass (red arrow) in the left upper outer quadrant with ipsilateral level 1 axillary lymphadenopathy (dotted yellow arrow). Axial MIP (c) and axial T1-weighted DCE with color (d) demonstrate the mass with neovascularization and mixed kinetics (red arrows). Biopsy depicted invasive carcinoma. Findings are consistent with AJCC TNM stage IIB (T2N1M0). AJCC: American Joint Committee on Cancer; TNM: tumor, node, and metastasis; MLO: mediolateral oblique; MIP: maximum intensity projection; DCE: dynamic contrast-enhanced.

In TNM stage IIB, MRI will either demonstrate a tumor >20 mm but ≤50 mm (T2), with movable ipsilateral level 1/2 axillary lymphadenopathy (N1), or it will demonstrate a tumor >50 mm (T3) without ipsilateral axillary lymphadenopathy (N0). There will be an absence of distant metastasis in the MRI field of view (M0) (Figure 4).

TNM stage: IIIA (T0-2N2M0/T3N1-2M0)

**Figure 5:**
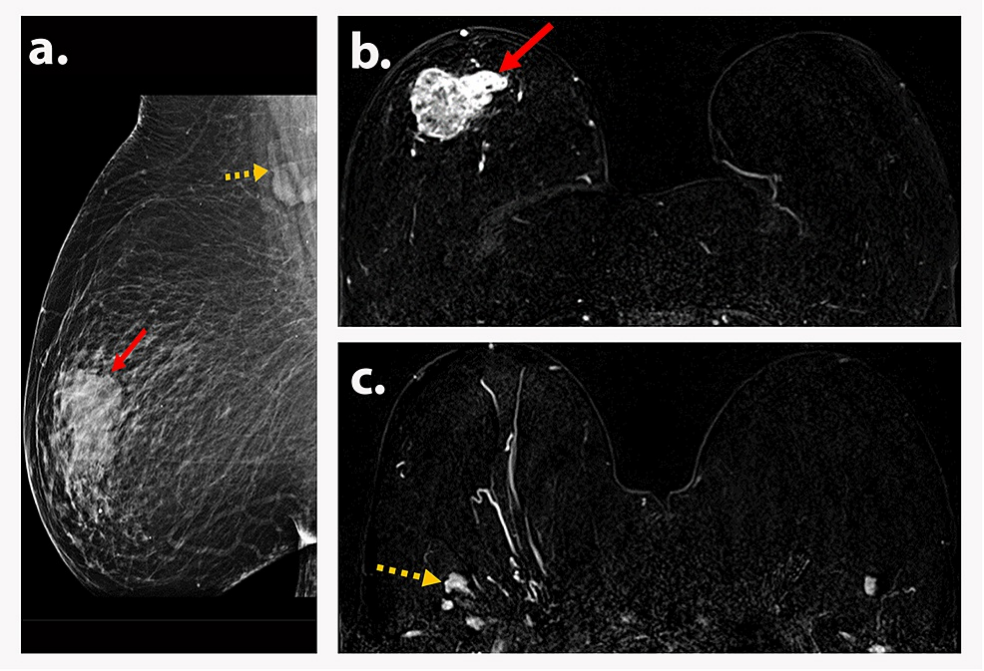
AJCC TNM stage IIIA (T3N1M0) A 40-year-old premenopausal woman presented with a palpable right breast mass. MLO of a screening mammography (a) shows a 6.6 cm irregular mass with indistinct and microlobulated margin (red arrow) in the superior right breast with axillary lymphadenopathy (yellow arrow). Axial T1-weighted DCE subtraction images (b and c) show a 6.6 cm irregular, heterogeneously enhancing mass (red arrow) in the upper outer quadrant of the right breast (red arrow) and associated level 1 axillary lymphadenopathy (dotted yellow arrow). Biopsy depicted invasive ductal carcinoma. Findings are consistent with AJCC TNM stage IIIA (T3N1M0). AJCC: American Joint Committee on Cancer; TNM: tumor, node, and metastasis; MLO: mediolateral oblique; DCE: dynamic contrast-enhanced.

In TNM stage IIIA, MRI can demonstrate no tumor or tumor ≤50 mm (T0-2) with ipsilateral matted level 1/2 axillary lymphadenopathy, or ipsilateral internal mammary nodes in the absence of axillary lymphadenopathy (N2). Alternatively, it is still considered stage IIIA, when MRI demonstrates a tumor >50 mm (T3) with either movable ipsilateral level 1/2 axillary lymphadenopathy (N1), or ipsilateral matted level 1/2 axillary lymphadenopathy, or ipsilateral internal mammary nodes in the absence of axillary lymphadenopathy (N2). There will be an absence of distant metastasis in the MRI field of view (M0) (Figure 5).

TNM stage: IIIB (T4N0-2M0)

**Figure 6:**
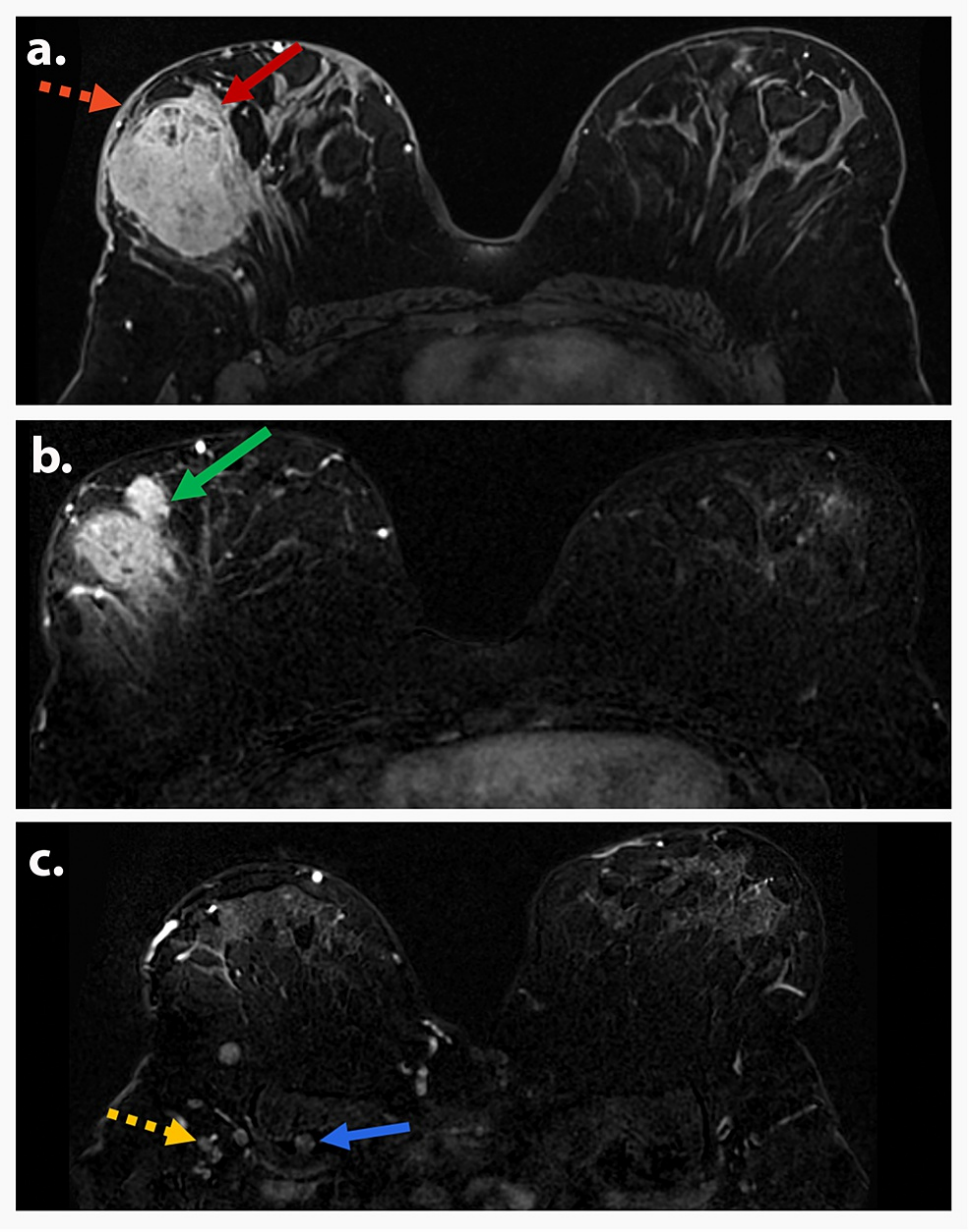
AJCC TNM stage IIIB (T4bN1M0) A 42-year-old female presented with a right breast mass, which on evaluation with a diagnostic mammogram, ultrasound, and subsequent biopsy was proven to be invasive ductal carcinoma. Axial postcontrast T1-weighted MR image (a) exhibits a 6.7 x 6 x 5.9 cm mass (red arrow) with heterogeneous enhancement in the lateral right breast with overlying thick and enhancing skin (dotted orange arrow), concerning for skin involvement (T4). Axial postcontrast T1-weighted subtraction MR image (b) shows a 2.2 x 1.9 x 1.7 cm enhancing mass (green arrow) at the anterior and inferior aspect of the index lesion at 9:00, 6 cm from the nipple. Axial postcontrast T1-weighted subtraction MR image (c) shows right axillary (dotted yellow arrow) and right sub-pectoral lymphadenopathy (blue arrow) (N1). Findings are consistent with AJCC TNM stage IIIB (T4bN1M0). AJCC: American Joint Committee on Cancer; TNM: tumor, node, and metastasis; MR: magnetic resonance; DCE: Dynamic contrast-enhanced.

In TNM stage IIIB, MRI can demonstrate a tumor with direct extension to the chest wall and/or skin (T4). Skin changes include ulceration and/or edema and/or satellite skin nodules. Lymphadenopathy can be absent (N0), or there can be movable ipsilateral level 1/2 axillary lymphadenopathy (N1), or ipsilateral matted level 1/2 axillary lymphadenopathy, or ipsilateral internal mammary nodes in the absence of axillary lymphadenopathy (N2). There will be an absence of distant metastasis in the MRI field of view (M0) (Figure 6).

TNM stage: IIIC (anyT0-4N3M0)

**Figure 7:**
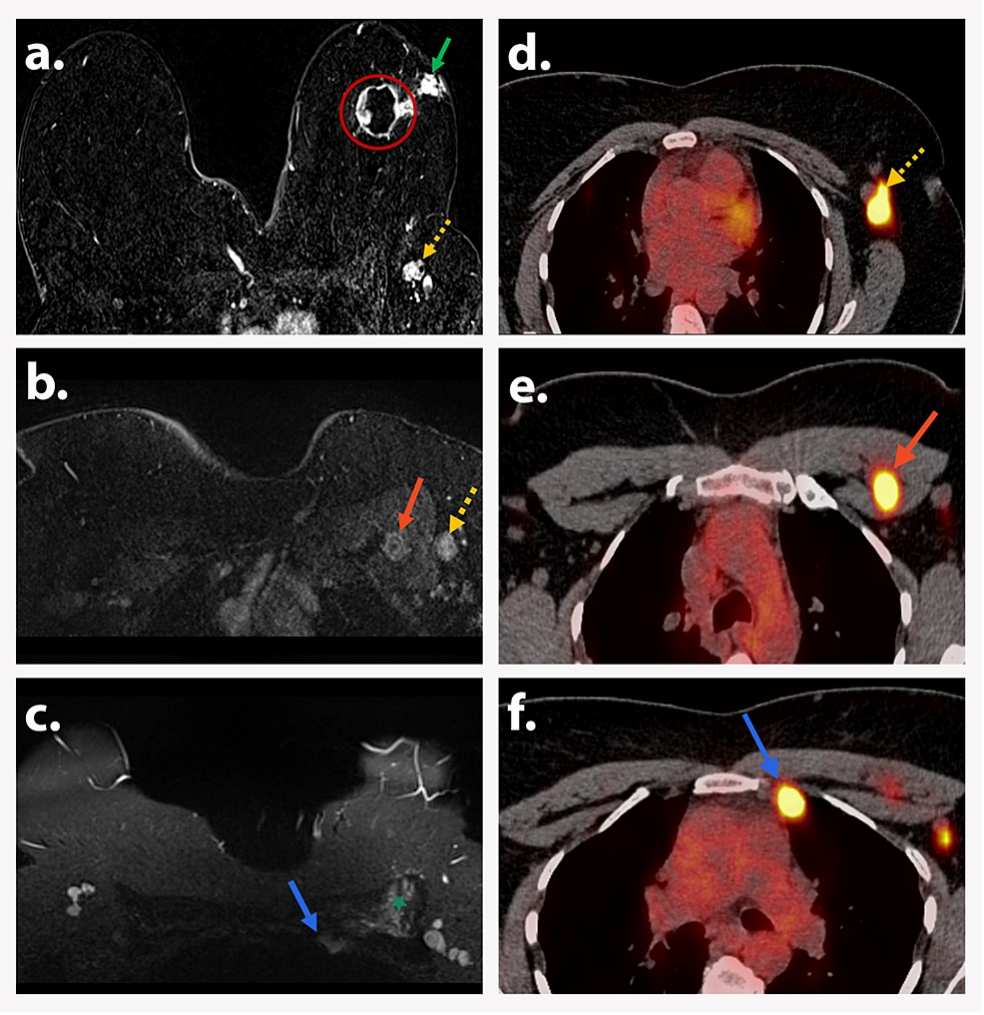
AJCC TNM stage IIIC (T2N3bM0) A 55-year-old postmenopausal woman presented with a new left breast mass. Axial postcontrast T1-weighted subtraction DCE image (a) shows a 3.5 cm irregular, heterogeneously enhancing mass with central necrosis (red circle) in the left breast, along with satellite focus (green arrow) and level 1 axillary lymphadenopathy (dotted yellow arrow). Axial T1-weighted DCE image (b) demonstrates level 1 (yellow arrow) and level 2 (orange arrow) left axillary lymphadenopathy. Axial STIR image (c) shows an abnormal ipsilateral internal mammary lymph node (blue arrow) deep and posterior to the pectoralis muscle (green asterisk). PET/CT images (d, e, and f) show FDG-avid left axillary level 1 (dotted yellow arrow, image d) and level 2 (orange arrow, image e), and internal mammary (blue arrow, image f) lymph nodes. Biopsy depicted invasive carcinoma. Findings are consistent with AJCC TNM stage IIIC (T2N3bM0). AJCC: American Joint Committee on Cancer; TNM: tumor, node, and metastasis; DCE: dynamic contrast-enhanced; STIR: short tau inversion recovery; PET/CT: positron emission tomography/computed tomography; FDG: fluorodeoxyglucose.

**Figure 8:**
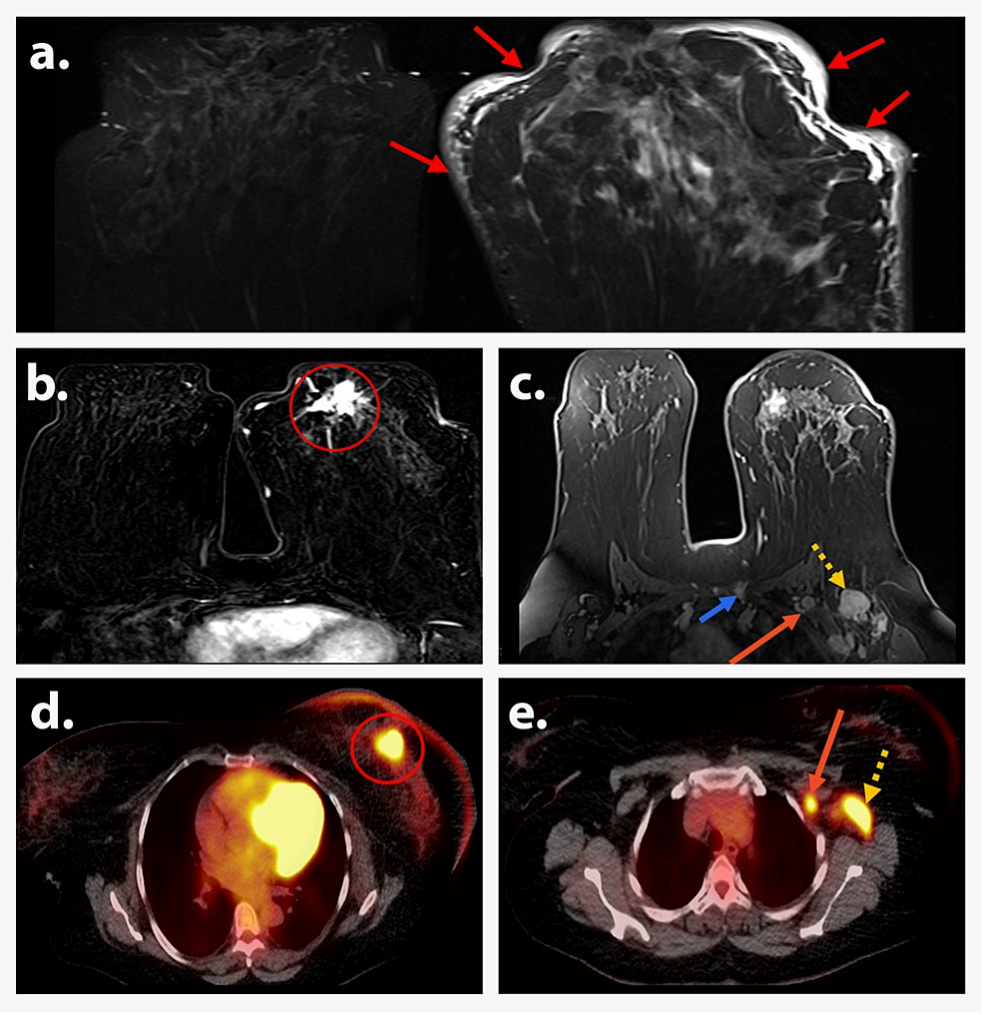
AJCC TNM Stage IIIC (T4dN3bM0) A 40-year-old premenopausal woman presented with left breast swelling and a palpable mass. Axial STIR image (a) shows left breast enlargement, diffuse edema, and skin thickening (red arrows). Axial T1-weighted subtraction DCE image (b) demonstrates a 1.8 cm irregular enhancing left retroareolar mass (red circle). Axial T1-weighted DCE image (c) shows level 1 (dotted yellow arrow) and level 2 (long orange arrow) left axillary lymphadenopathy and enlarged enhancing left internal mammary chain lymph node (short blue arrow). Axial PET/CT images (d and e) demonstrate FDG-avid skin thickening and edema and left retroareolar mass (red circle, image d), along with level 1 (dotted yellow arrow, image e) and level 2 (long orange arrow, image e). Biopsy depicted infiltrating ductal carcinoma. Findings are consistent with AJCC TNM Stage IIIC (T4dN3bM0). AJCC: American Joint Committee on Cancer; TNM: tumor, node, and metastasis; STIR: short tau inversion recovery; DCE: dynamic contrast-enhanced; PET/CT: positron emission tomography/computed tomography; FDG: fluorodeoxyglucose.

In TNM stage IIIC, MRI can demonstrate no tumor or tumor of any size, even those with direct extension to the chest wall and/or skin (T0-4). It can demonstrate either metastasis in ipsilateral infraclavicular lymph nodes with or without level 1/2 axillary lymph nodes involvement, or ipsilateral internal mammary with ipsilateral level 1/2 axillary lymph nodes, or ipsilateral supraclavicular lymph nodes with or without axillary or internal mammary lymph node involvement (N3). There will be an absence of distant metastasis in the MRI field of view (M0). TNM stage IIIC includes tumors of any T staging. MRI is able to optimally depict the extent of the tumor and even advanced T staging, for example, breast enlargement, diffuse skin thickening, and edema (Figures 7, 8).

TNM stage: IV (anyT0-4anyN0-3M1)

**Figure 9:**
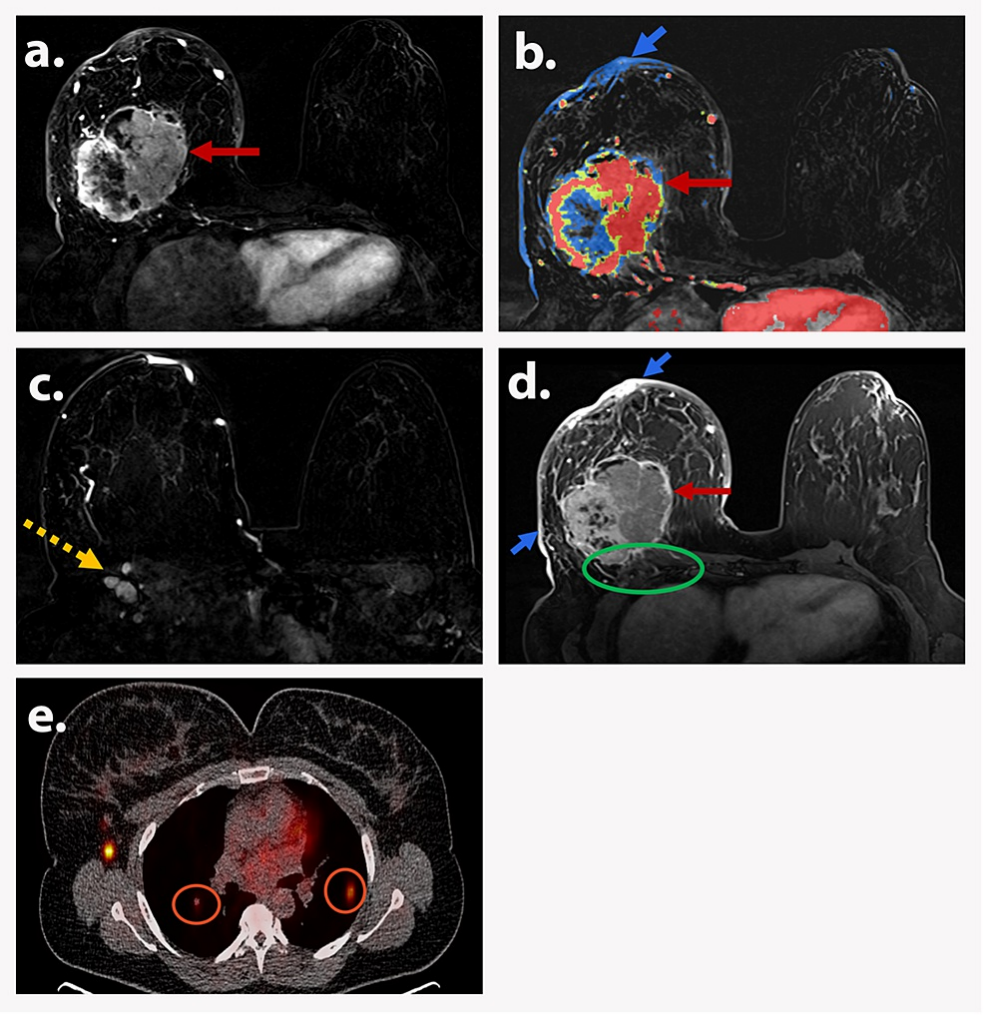
AJCC TNM Stage IV (T4cN1M1) A 46-year-old premenopausal woman presented to the emergency room with skin rash, swelling, and suspected abscess of the right breast. Axial T1-weighted subtraction DCE image (a) and corresponding color-coded map (b) show a 10.5 cm round, heterogeneously enhancing mass (red arrows) in the right breast with mixed kinetics. Evidence of diffuse skin thickening and enhancement (short blue arrow) is also noted. Axial T1-weighted subtraction DCE image (c) shows level 1 right axillary lymphadenopathy (dotted yellow arrow). Axial postcontrast T1-weighted image (d) shows diffuse right breast skin thickening (short blue arrows) and the mass (red arrow) abutting the pectoralis major muscle with obliteration of the fat plane and enhancement of the muscle (green circle). Axial PET/CT image shows bilateral FDG-avid pulmonary metastasis (orange circles). Biopsy depicted invasive carcinoma. Findings are consistent with AJCC TNM stage IV (T4cN1M1). AJCC: American Joint Committee on Cancer; TNM: tumor, node, and metastasis; DCE: dynamic contrast-enhanced; PET/CT: positron emission tomography/computed tomography; FDG: fluorodeoxyglucose.

In TNM stage IV, MRI can demonstrate any tumor (T0-4) and nodal (N0-3) staging; however, the key feature is distant metastasis (M1). Breast MRI may be able to identify some cases with distant metastasis, especially to the lung, bones, and occasionally the liver (Figure 9).

## Discussion

Key information to provide in the breast MRI report

Reporting a breast MRI for breast cancer evaluation should start with understanding the precise clinical question. A detailed description of imaging findings should follow using the recognized BI-RADS descriptors. The findings should be based on the TNM staging, keeping in mind the key findings that will alter treatment as listed below (Tables 6-8). Finally, a concise impression with a final assessment score and a clear recommendation for management will complete the MRI report [1,11,12].

**Table 6:** T staging AP: anteroposterior; CC: craniocaudal; TRV: transverse.

Tumor or non-mass enhancement size: AP, CC, and TRV dimensions
Maximum dimension of the largest tumor is used
Location using quadrants or o’clock position and distance from the nipple
Depth: anterior, middle, posterior
Additional foci are important for surgical planning but will not affect staging
Skin or nipple involvement
Pectoralis muscle involvement
Multifocal disease: two lesions within the same quadrant
Multicentric: two lesions in different quadrants
Chest wall involvement: intercostal muscles, ribs, or serratus anterior muscle

**Table 7:** N staging

Regional lymph nodes: ipsilateral axillary, internal mammary, and supraclavicular
Axillary lymph nodes are divided into levels using pectoralis minor muscle as a reference
Level I: inferior to the inferolateral border of the pectoralis minor
Level II: posterior to and between medial and lateral borders of pectoralis minor
Level III: medial to the superior border of the pectoralis minor, including infraclavicular lymph nodes

**Table 8:** M staging

M1 - contralateral breast tumor
M1 - Distant metastasis in visualized field of view - lung, bones, and liver

Advantages of using breast MRI for staging

Breast MRI plays an important role in breast cancer screening and diagnosis, based on the current indications, as per the ACR practice parameters [7]. It has traditionally been used as an adjunct modality for the evaluation of complex breast cancer cases or for providing answers to clinical problems when they are not apparent on initial imaging with mammogram and ultrasound. However, primarily because of its excellent ability to define the extent of disease, breast MRI is increasingly proving to be the key non-invasive tool for staging breast cancer, determining treatment options, and preparing for the most optimal surgery [13].

Contrast-enhanced breast MRI has demonstrated utility in identifying additional tumor foci and the extent of disease in patients with known breast cancer. This is especially useful with invasive lobular carcinoma, which is difficult to evaluate on mammography. If neoadjuvant chemotherapy is being considered for breast cancer treatment, a pre-chemotherapy MRI permits the identification of disease extent and allows for more accurate post-treatment evaluation [14].

The best non-invasive tool for assessing accurate tumor size is breast MRI. Tumor size can be underestimated by mammography and ultrasound, but the size of the tumor at histology is not significantly different from that on MRI [15]. In breast MRI, both breasts are imaged at the same time, which helps evaluate contralateral disease. It also assesses for multifocal or multicentric disease, which does alter surgical management [16]. Generally, for multifocal disease (i.e., disease occupying the equivalent of one quadrant of the breast or less), breast conservation surgery can be attempted. On the other hand, for cases of multicentric disease (i.e., disease occupying the equivalent of more than one quadrant of the breast), mastectomy is often required [17]. Contrast-enhanced MRI also allows for simultaneous assessment of the involvement of the pectoral muscle and chest wall. Pectoral muscle involvement does not affect staging but alters surgical management, whereas chest wall involvement affects both. Breast MRI allows for the evaluation of levels 1, 2, and 3 axillary and internal mammary lymph nodes, which not only affects staging and treatment planning but is also the most important deterministic factor for the prognosis of breast cancer. Finally, it is imperative to look for and report extra-mammary findings in the lungs, bones, and visualized liver, especially if they are concerning for distant metastasis [12].

Limitations of breast MRI

MRI is an expensive test and also takes longer to perform than other breast imaging modalities. MRI is contraindicated in patients with non-MRI-compatible cardiac pacemakers, neurostimulators, and certain aneurysm clips, and prosthetic cardiac valves.

MRI is a highly sensitive and specific modality for breast cancer; however, it has its own limitations. The routine use of MRI in pre-operative breast cancer staging is debated, because it has not been shown to improve patient outcomes, including recurrence or mortality. Image-guided biopsy is required to prove multifocal, multicentric, or contralateral cancer when additional suspicious findings are picked up on MRI in a patient with breast cancer.

## Conclusions

Radiologists should be familiar with the latest 8th edition of the AJCC TNM staging system for breast cancer. Mammogram, ultrasound, and MRI are all important imaging modalities with specific roles in the evaluation and staging of breast cancer. By applying knowledge of the staging and imaging findings to individual cases, radiologists can play a pivotal role in the decision-making process, alongside breast surgeons and oncologists.

Breast MRI has been used traditionally as a problem-solving tool in breast cancer treatment planning. However, the use of breast MRI as a breast cancer staging tool can provide key information that is critical to both patient management and prognosis.
